# Supplementary material for: Structural distortion and electron redistribution in dual-emitting gold nanoclusters
Source: Nat Commun. 2020 Jun 9;11:2897. doi: 10.1038/s41467-020-16686-8 (PMC7283347; doi:10.1038/s41467-020-16686-8)
Supplement: Supplementary file 1 — Supplementary Information [file 41467_2020_16686_MOESM1_ESM.pdf]

Supplementary Information

Structural Distortion and Electron Redistribution in Dual-emitting Gold Nanoclusters

Li et al.

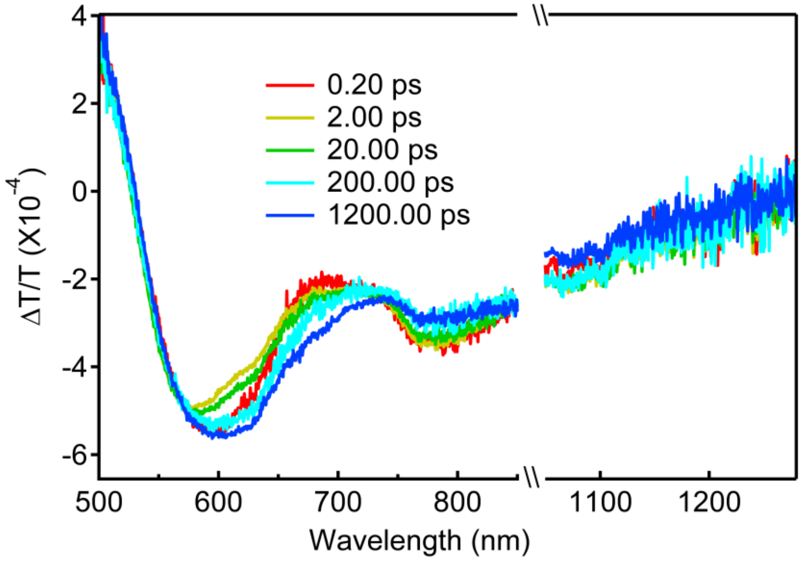

Supplementary Figure 1. fs-TA spectra of Au_{24} at selected time delays (solvent: DCM).

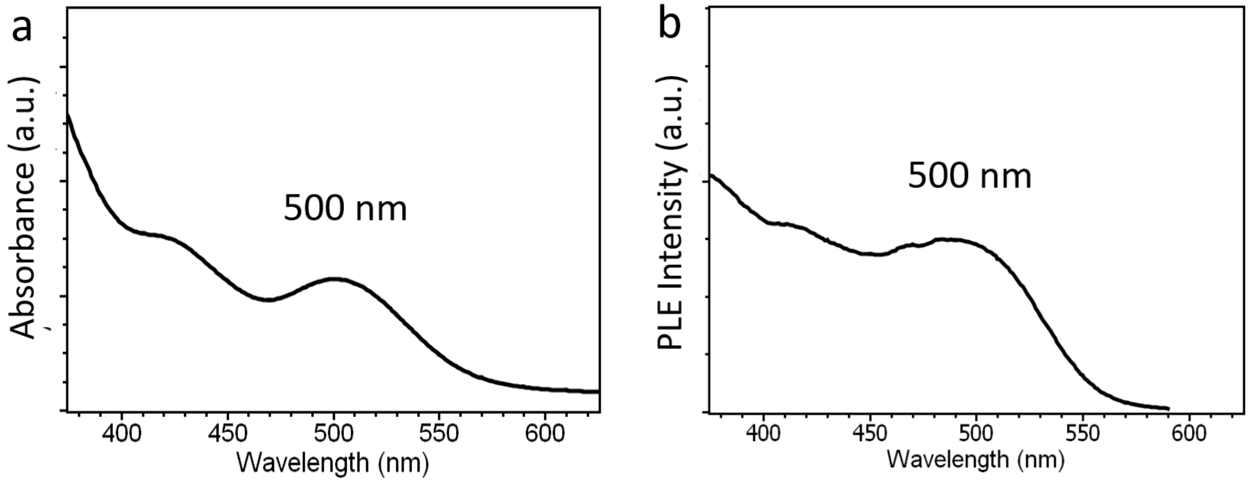

Supplementary Figure 2. (a) UV-vis absorption and (b) PL excitation (detected at the wavelength of 600 nm) spectra of Au_{24} in hexane.

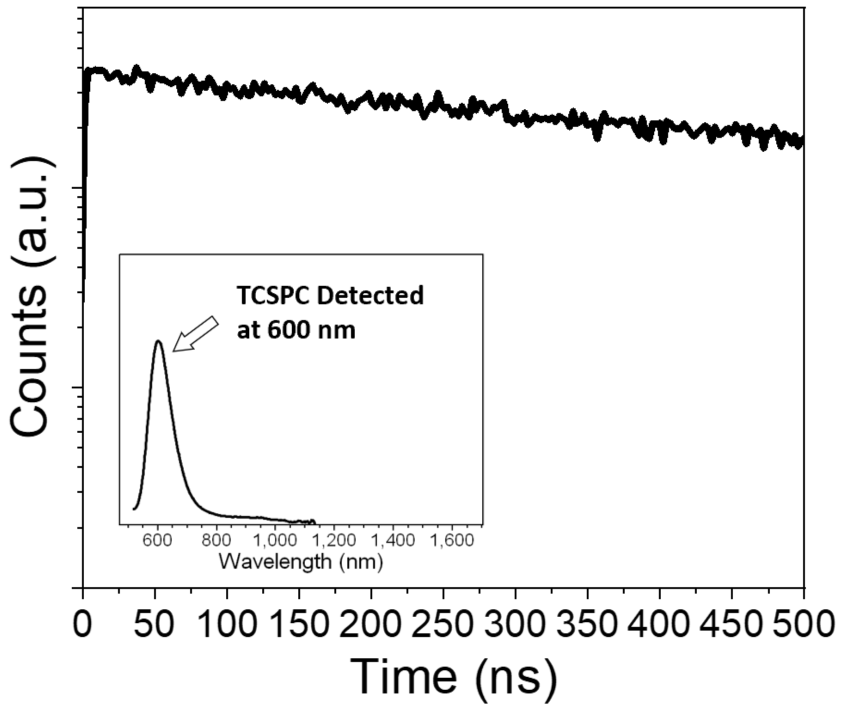

Supplementary Figure 3. TCSPC trajectory of Au_{24} in butanol. Inset is the PL of Au_{24} in butanol, the TCSPC data was detected at 600 nm.

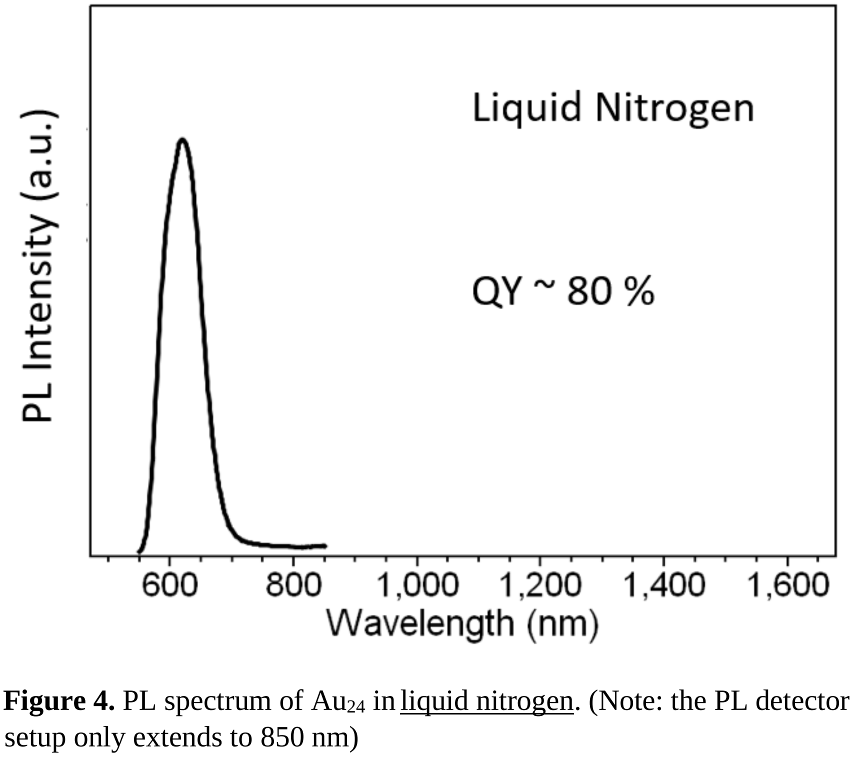

Supplementary Figure 4. PL spectrum of Au_{24} in liquid nitrogen. (Note: the PL detector in our in-home low-temperature setup only extends to 850 nm)

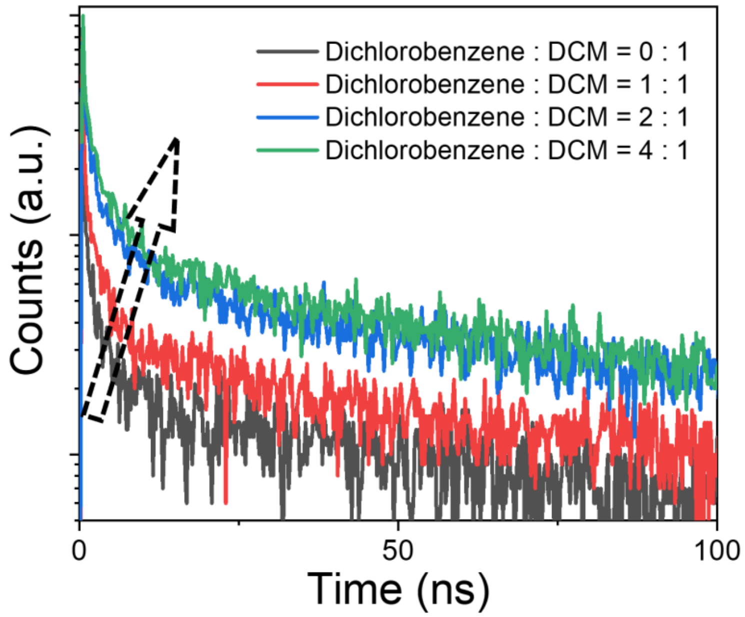

Supplementary Figure 5. TCSPC trajectory of the PL I at 650 nm of Au_{24} in different ratios of DCM/1,2-dichlorobenzene.

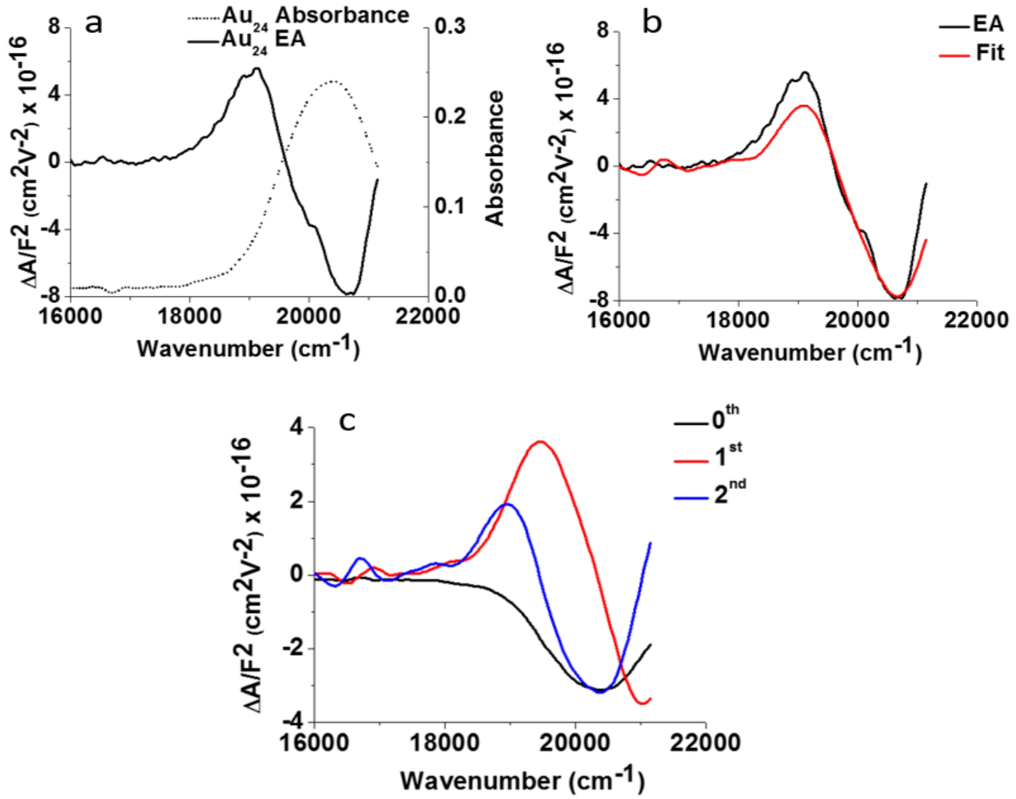

Supplementary Figure 6. Stark spectroscopy measurements on Au_{24}. (a) Absorbance (dashed line) and electroabsorption (solid). (b) EA (black) and fit (red). (c) EA fit broken into 0th derivative, 1st derivative, and 2nd derivative.

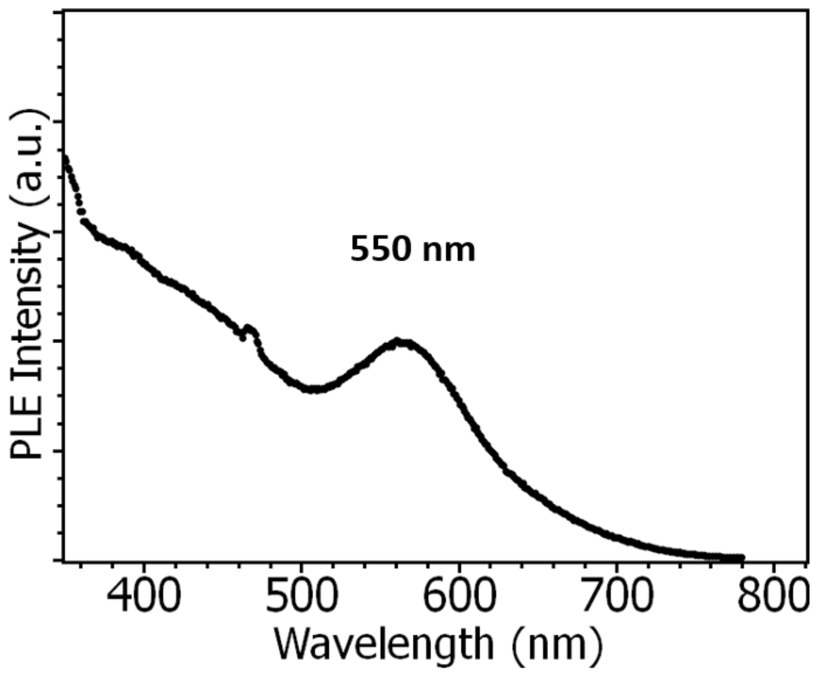

Supplementary Figure 7. PL excitation spectrum of the $Au_{14}Cd_1$ in DCM.

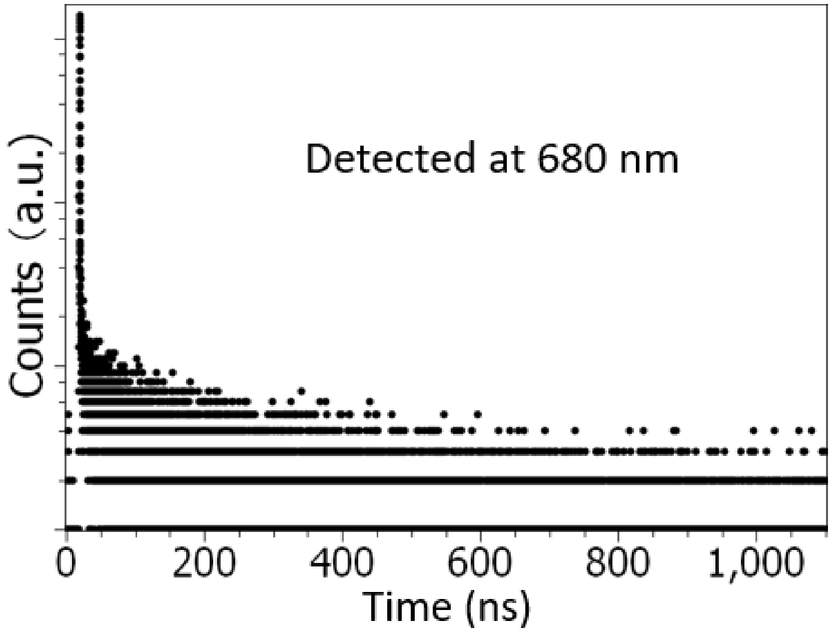

Supplementary Figure 8. TCSPC trajectory of the $Au_{14}Cd_1$ in DCM detected at 680 nm.

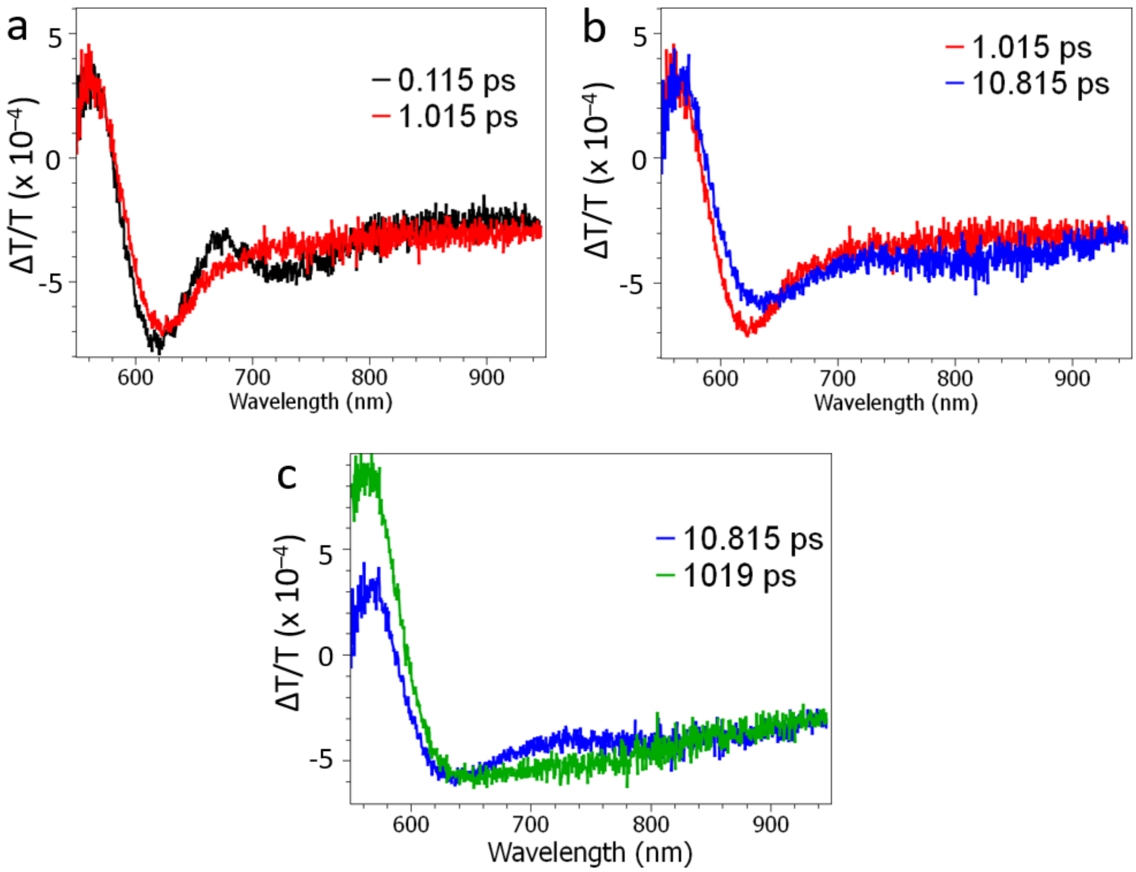

Supplementary Figure 9. fs-TA spectra of $Au_{14}Cd_1$ at typical time delays in DCM pumped at 550 nm. (a) from 0–1 ps. (b) from 1–10 ps. (c) from 10–1000 ps.

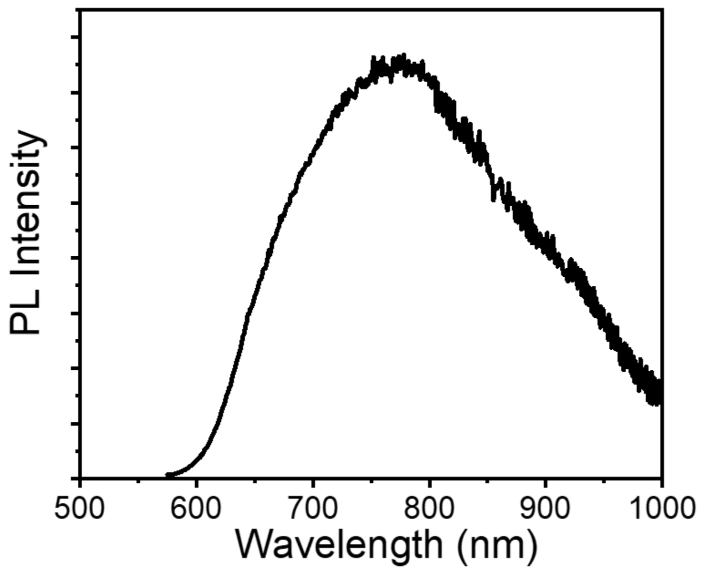

Supplementary Figure 10. PL Spectrum of $Au_{14}Cd_1$ in hexane.

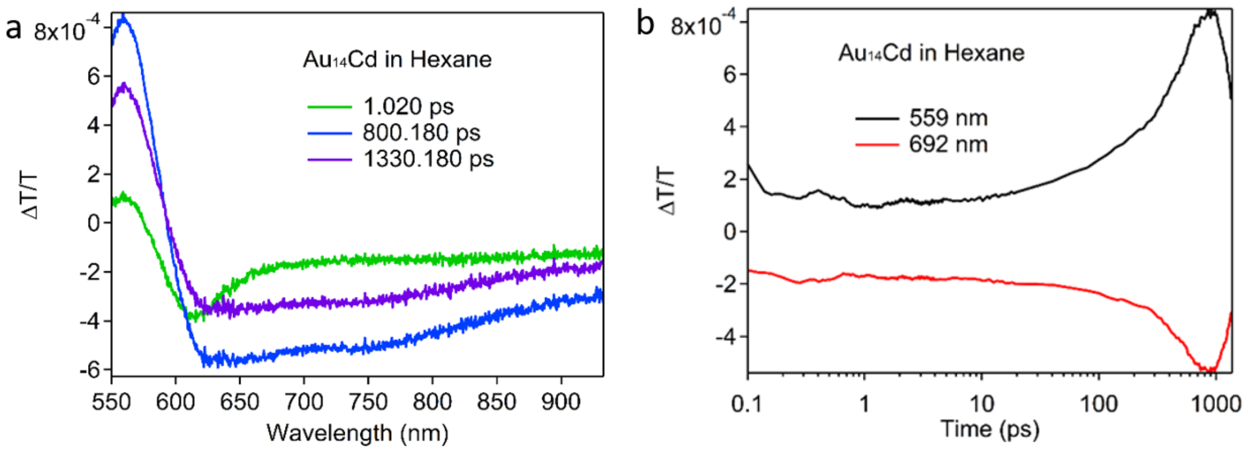

Supplementary Figure 11. (a) fs-TA spectra of $Au_{14}Cd_1$ in hexane at typical time delays. (b) Selected kinetic traces at typical wavelengths.

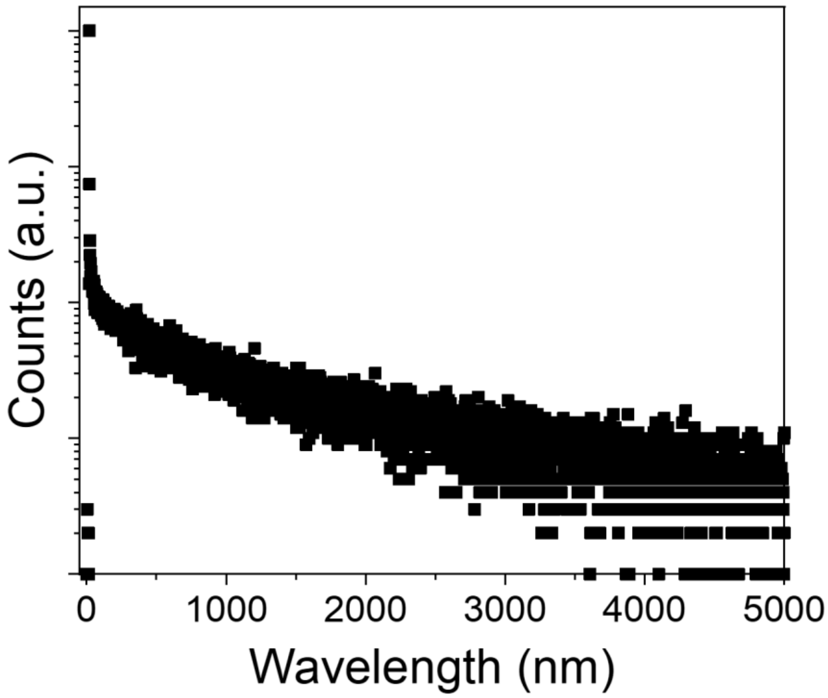

Supplementary Figure 12. TCSPC trajectory of the Au_{24}' in DCM detected at 800 nm.

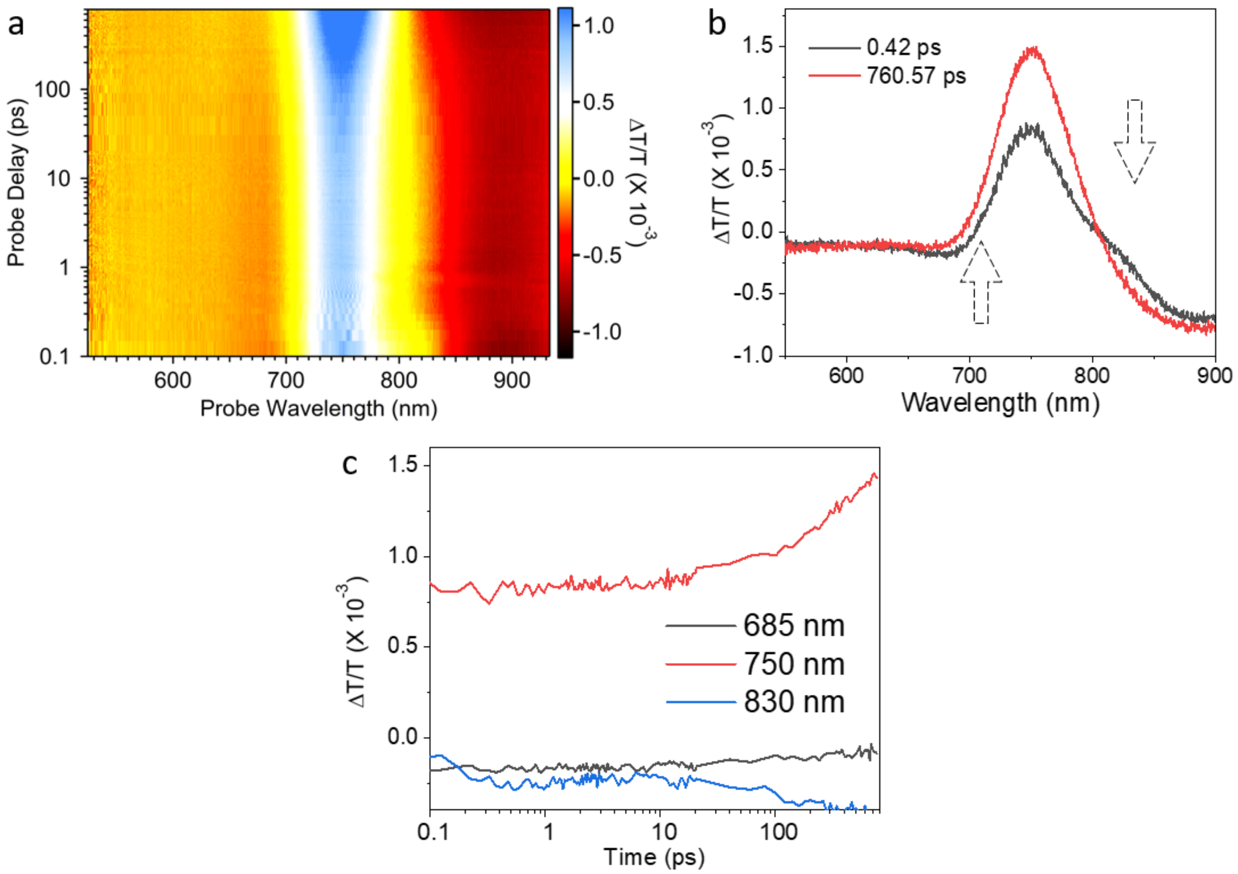

Supplementary Figure 13. (a) fs-TA spectra of Au_{24}' pumped at 520 nm which shows the ΔT/T at all time-delays between 500–900 nm. (b) fs-TA spectra of Au_{24}' at typical time delays. (c) Selected kinetic traces at typical wavelengths.

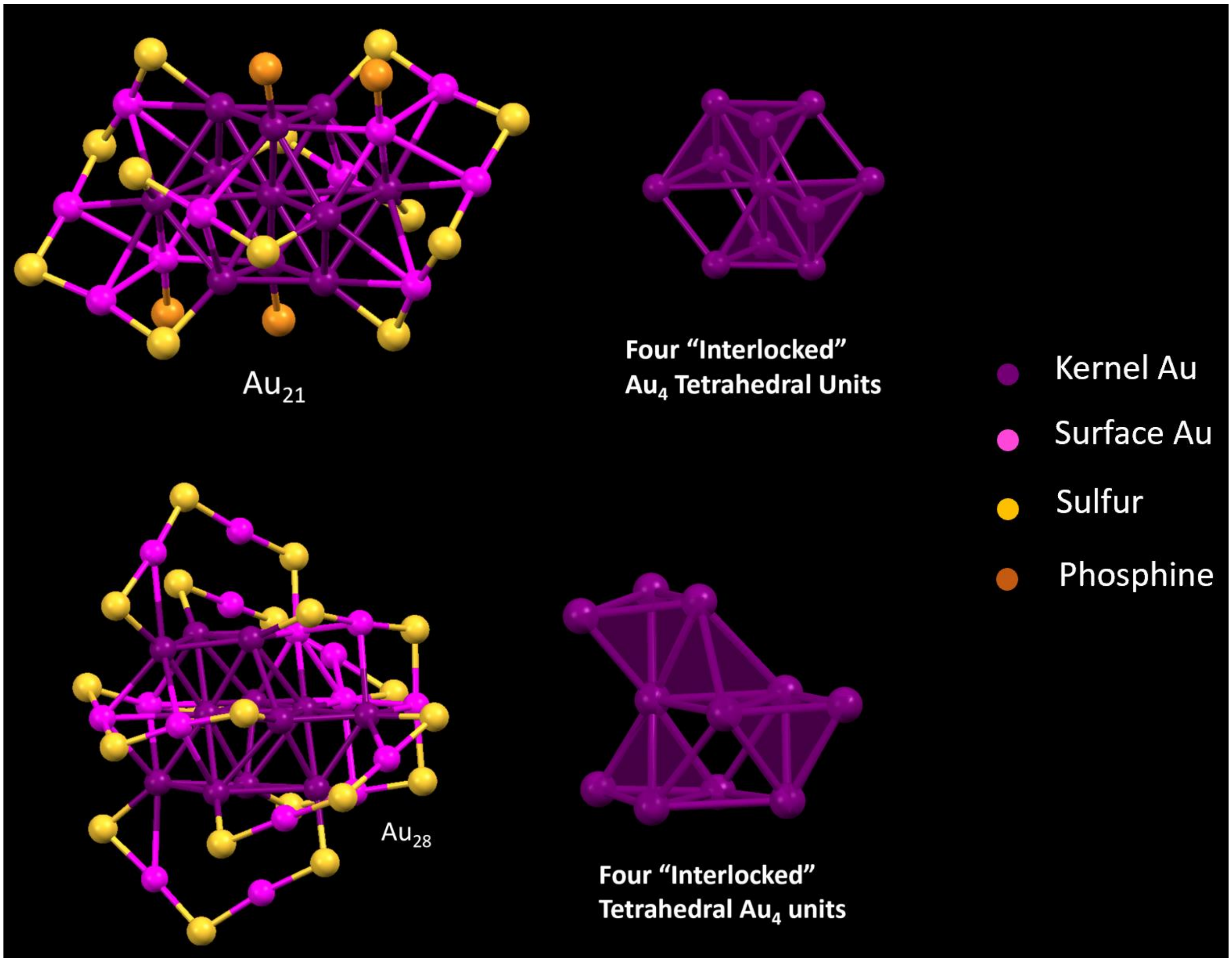

Supplementary Figure 14. Atomic structure of Au_{21} and Au_{28} determined by single-crystal X-ray diffraction.[1,2] In these two nanoclusters, the four tetrahedra share vertex and sides and thus being interlocked. The "movement" and structure-distortion of the Au_4 units have been hindered.

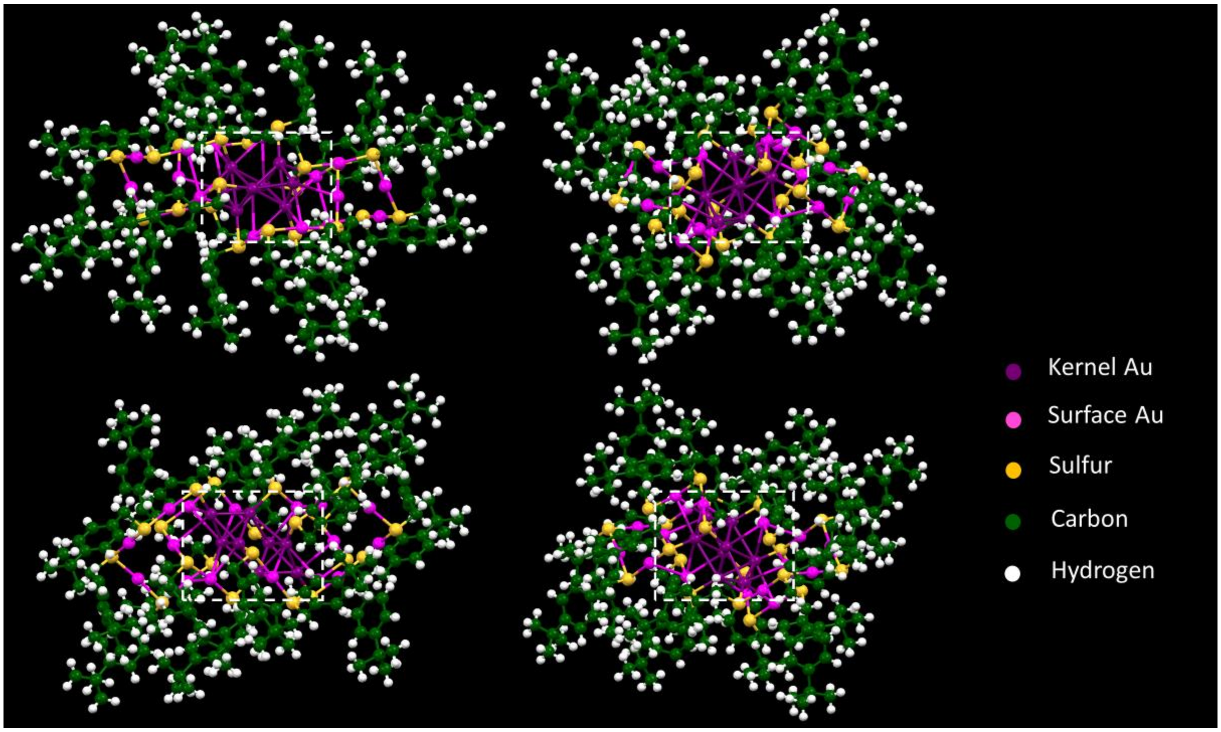

Supplementary Figure 15. The “open” Au_8 kernel of Au_{24} from different viewing directions.

Supplementary Table 1. Solvent dependence of the PL from Au_{24}.

Solvent	PL I	Polarity Index	Viscosity (cp *r.t.*)
DCM	670 nm	3.1	0.43
Toluene	650 nm	2.4	0.59
Hexane	610 nm	0.1	0.31
DCM	670 nm	3.1	0.43
1,2-Dichlorobenzene	660 nm	2.7	1.32
1-Butanol	600 nm	3.9	2.95
1-Octanol	590 nm	3.4	10.6

Supplementary References

1. Li, Q.; Luo, T.-Y.; Taylor, M. G.; Wang, S.; Zhu, X.; Song, Y.; Mpourmpakis, G.; Rosi, N. L.; Jin, R. Molecular “Surgery” on a 23- Gold-Atom Nanoparticle. *Sci. Adv.* **2017**, *3*, No. e1603193.

2. Zeng. C.; Li, T.; Das, A.; Rosi, N. L.; Jin, R.; *J. Am. Chem. Soc.* **2013**, *135*, 27, 10011-10013
